# Application of a modified osteotomy and positioning integrative template system (MOPITS) based on a truncatable reconstruction model in the precise mandibular reconstruction with fibula free flap: a pilot clinical study

**DOI:** 10.1186/s12903-023-03596-6

**Published:** 2023-11-08

**Authors:** Qing Sun, Zhihui Zhu, Fanhao Meng, Ruiqi Zhao, Xing Li, Xiao Long, Yansheng Li, Haitao Dong, Tao Zhang

**Affiliations:** 1 Department of Plastic Surgery, Peking Union Medical College &, Chinese Academy of Medical Science, Beijing, China; 2 Department of Stomatology, Peking Union Medical College &, Chinese Academy of Medical Science, Beijing, China; 3 Department of Stomatology, Beijing Friendship Hospital, Capital Medical University, Beijing, China; 4 Beijing University of Technology, Beijing, China

**Keywords:** Guide plate, Fibula flap, CAD/CAM, Virtual surgical planning, 3D printing

## Abstract

**Background:**

Mandibular defects can greatly affect patients' appearance and functionality. The preferred method to address this issue is reconstructive surgery using a fibular flap. The current personalized guide plate can improve the accuracy of osteotomy and reconstruction, but there are still some problems such as complex design process and time-consuming. Therefore, we modified the conventional template to serve the dual purpose of guiding the mandible and fibula osteotomy and facilitating the placement of the pre-bent titanium.

**Methods:**

The surgery was simulated preoperatively using Computer-Aided Design (CAD) technology. The template and truncatable reconstruction model were produced in the laboratory using 3D printing. After pre-bending the titanium plate according to the contour, the reconstruction model was truncated and the screw trajectory was transferred to form a modified osteotomy and positioning integrative template system (MOPITS). Next, the patient underwent a composite template-guided vascularized fibula flap reconstruction of the mandible. All cases were reviewed for the total operative time and accuracy of surgery.

**Results:**

The procedures involved 2–4 fibular segments in 15 patients, averaging 3 fibular segments per procedure. The osteotomy error is 1.01 ± 1.02 mm, while the reconstruction angular error is 1.85 ± 1.69°. The preoperative and postoperative data were compared, and both *p* > 0.05. During the same operation, implant placement was performed on four patients, with an average operative time of 487.25 ± 60.84 min. The remaining malignant tumor patients had an average operative time of 397.18 ± 73.09 min. The average postoperative hospital stay was 12.95 ± 3.29 days.

**Conclusions:**

This study demonstrates the effectiveness of MOPITS in facilitating precise preoperative planning and intraoperative execution of fibula flap reconstruction. MOPITS represents a promising and reliable tool for reconstructive surgery, particularly for inexperienced surgeons navigating the challenges of mandible defect reconstruction.

**Supplementary Information:**

The online version contains supplementary material available at 10.1186/s12903-023-03596-6.

## Introduction

As a crucial element in the skeletal framework of the lower face, the mandible plays an important role in maintaining the natural facial appearance. Benign and malignant tumors, infections, and trauma in the maxillofacial region may cause mandibular segmental defects [1]. These defects can lead to facial deformities and impair oral functions, resulting in psychological burden and mental stress.

The fibula free flap (FFF) has become the preferred approach for addressing mandibular defects, mainly because of its ability to be easily harvested, carved with precision, and produce satisfactory functional outcomes [2]. Other vascularised flaps utilised for reconstruction of the maxillofacial complex comprise the iliac crest, scapula, radial forearm and latissimus-serratus-rib flaps. Nonetheless, for segmental mandibular reconstruction, these free flaps have been replaced essentially by FFF [3]. Firstly, the flap can be up to 20–25 cm in length, making it suitable for repairing significant jaw defects. Additionally, the fibula is a tough bone with a cortical to medullary bone ratio of roughly 1:1, making it ideal for denture implants. The fibula is supplied by both periosteum and bone marrow with a substantial blood supply, allowing for flexible shaping and strong resistance to infection. The flap possesses a lengthy vascular pedicle and is capable of carrying one or more skin islands to remedy complex maxillofacial defects, serving as an "observation window" for monitoring blood supply [4].

Achieving successful outcomes requires a high standard for head and neck reconstruction surgeons. Digital technologies, such as computer-aided design/manufacturing (CAD/CAM), have increasingly used vascularized fibula flaps in mandibular reconstruction [5]. CAD/CAM technology has greatly enhanced the precision of mandibular osteotomies compared to empirical methods [6]. However, even with preoperative virtual surgical planning, it is difficult to adjust the titanium plate to the desired position during surgery without anatomical landmarks. Particularly for junior doctors, positioning the titanium plate and fibula segments can be challenging and time-consuming, potentially leading to decreased accuracy in the final result.

In recent years, researchers have been actively exploring personalized templates. Some surgeons have implemented pre-bent titanium plate techniques and screw trajectory transfer techniques to improve the precision of mandibular reconstruction procedures based on preoperative designs [7, 8]. The design of the positioning holes on the guide plate can simplify the procedure. Most studies on positioning holes have primarily focused on the jaw guide [9], while limited studies on the fibular guide plate exist. Furthermore, these studies require multiple scans, additional shaping guides or personalized titanium plates [10]. Nevertheless, the design process for most of these methods can be complex and time-consuming, which hinders the widespread adoption of these techniques in primary care hospitals.

Therefore, we modified the conventional template to serve the dual purpose of guiding the mandible and fibula osteotomy and facilitating the placement of the pre-bent titanium without any added costs or increased fabrication complexity. This study uses a vascularized fibula flap to present a modified osteotomy and positioning integrative template system (MOPITS) for mandibular reconstruction. The MOPITS system uses the truncatable reconstruction model and screws trajectory transfer technology.

## Materials and methods

### Patients

A total of 15 patients, consisting of 11 male and 4 female cases, who underwent mandible reconstruction using a vascularized fibula flap at the Department of Oral Maxillofacial Surgery, Peking Union Medical College Hospital, between July 2020 and January 2023, were included in the study. The age of the patients ranged from 12 to 79 years, with a mean age of 55.79 years. The inclusion criteria were as follows: 1) malignant neoplasm involving the mandible; 2) patient and family consent for utilizing MOPITS during the procedure and signed an informed consent form; 3) MOPITS was applied to perform mandibular reconstruction surgery without the use of other digital techniques. Exclusion criteria for patients were as follows: 1) Underwent multiple frozen sections due to positive margin intraoperatively 2) Underwent mandible reconstructive procedures previously; 3) Simultaneous multiple flap surgery; 4) incomplete availability of preoperative and postoperative data for patients.

Squamous cell carcinoma was the most prevalent malignancy in this cohort, comprising 73.33% of the cases. Mandibular defects were evaluated using the Brown classification system [11, 12]; the results showed that 60% of cases were classified as category II, 26.67% as category III, 6.67% as category IV, and 6.67% as category I. In three cases, the defects involved the condyle. Patient characteristics, including age at the time of reconstruction, gender, diagnosis, defect classification, and the number of fibula segments utilized, were retrieved from medical records (Table 1).

**Table 1:** Demographic data and clinical characteristics of the 15 patients

Patient Number	Age	Gender	Pathological Diagnosis	Brown Classification	Layers of fibular flap	Fibula Segments
1	49	M	SCC	I	2	2
2	22	M	OS	IVc	2	4
3	12	F	RMS	IIc	2	3
4	43	M	SCC	III	2	4
5	72	M	SCC	II	2	4
6	48	M	MEC	II	2	3
7	47	M	SCC	IIc	2	3
8	79	M	MEC	III	1	2
9	63	F	SCC	II	2	2
10	79	F	SCC	III	1	2
11	54	M	SCC	II	2	3
12	68	M	SCC	II	2	4
13	70	M	SCC	III	2	3
14	64	M	SCC	II	2	3
15	69	F	SCC	II	2	3

*SCC* Squamous carcinoma, *OS* Osteosarcoma, *RMS* Rhabdomyosarcoma, *MEC* Mucoepidermoid carcinoma

### Preoperative design of personalized surgical templates and truncatable reconstruction model using computer-aided design (CAD)

**Fig. 1:**
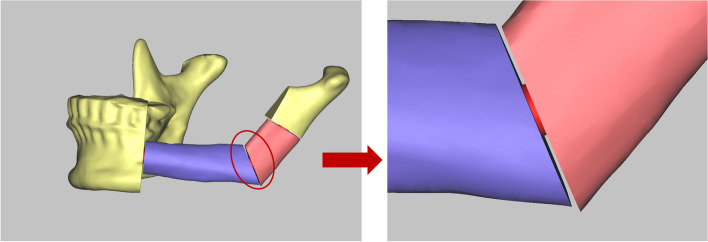
Preoperative design of truncatable reconstruction model. The picture pointed to the right by the arrow is the connecting post detail diagram of the truncatable model

All patients included in the study underwent several imaging examinations to evaluate the relevant vasculature, including cone beam computed tomography (CBCT, 3D Exam, KAVO, Germany), head routine scan CT (Siemens Healthcare, Forchheim, Germany), and CT angiography of lower extremities. A portable Doppler ultrasonic blood flow survey meter (Hadeco, ES-1000SPM, Japan) was performed to assess the location of the peroneal artery perforator. Three-dimensional CT data were saved in the Digital Imaging and Communications in Medicine (DICOM) and then processed using Proplan CMF 3.0 (Materialize, Belgium). If there were any distortions of the mandible caused by the tumor, the "mirror" function replicates the corresponding contralateral anatomical structure. The complete mandibular and fibula are simulated and reconstructed using the segmentation function. The extent of the cut is determined by combining the patient's CT images and the characteristics of the lesion. A simulated cut is then performed on a constructed model of the diseased mandible. Finally, the fibula segment with the optimal length and angle is identified. The fibula segment is adjusted according to the position of the peroneal artery skin-piercing branch. A connecting post, measuring 5 mm in diameter and 0.5 mm in height, was created at the joint of the bone segments to offer a workspace with minimal errors for the saw blade to produce a truncatable model (Fig. 1).

The 3-Matic 7.0 (Materialize, Belgium), Geomagic Studio 2017(Geomagic, America), and Magics 20 (Materialize, Belgium) software was used to design the osteotomy template for the mandibular lesion and the osteotomy template for the fibula. The guide plate utilized in this study incorporates a 2.5 mm thick guide slot and a width of 0.5 mm at the central cutting line, effectively stabilizing the cutting direction.

### Fabrication of MOPITS in the laboratory: intraoperative situation simulation using a truncatable model for screw trajectory transfer

**Fig. 2:**
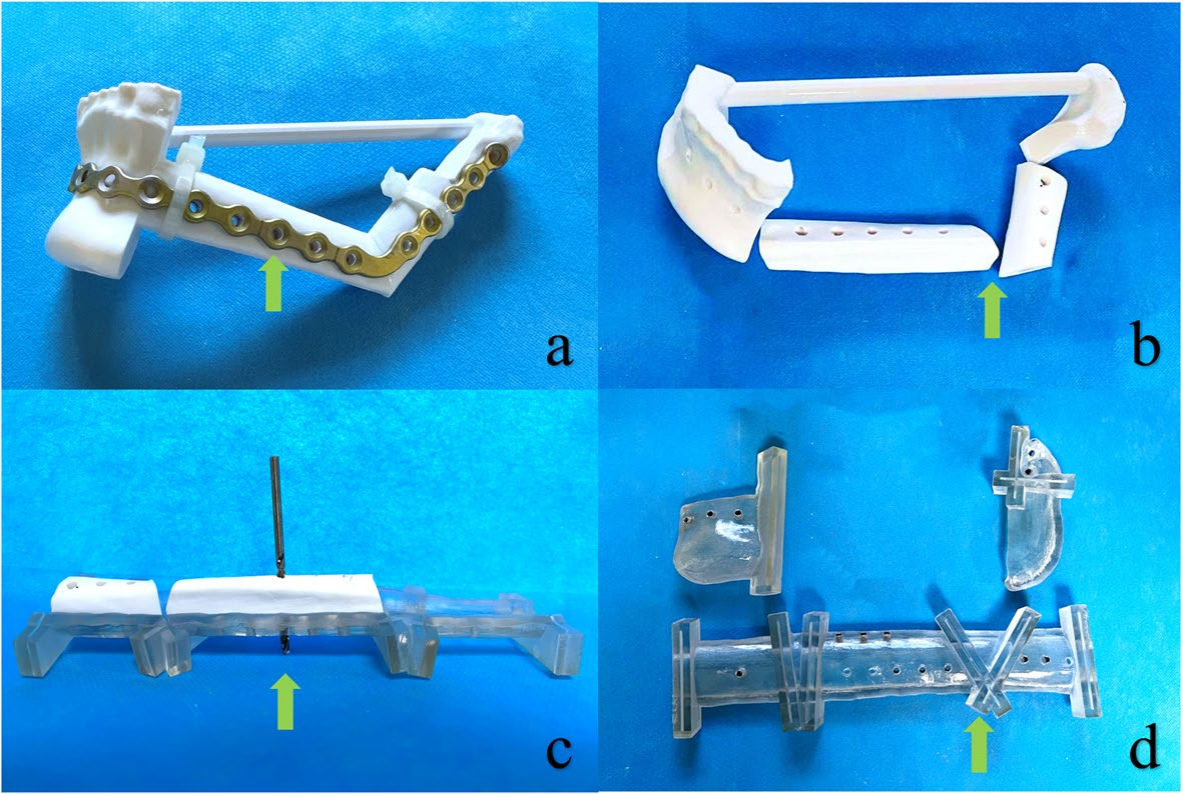
The process of making MOPITS: **a** Fixing the pre-bent titanium plate to the 3D printed reconstruction model. The arrow points to the titanium plate. **b** The reconstructed model after drilling the holes for titanium plate fixation was truncated into the mandible part and the fibula part. The arrow points to the truncated connection part. **c** Place the truncated model on the template and transfer the screw trajectory to the template. The arrow points to the drill. **d** MOPITS. The arrow points to the guide slot

In this study, we utilized 3D printers (FORM 3BL, Formlabs, America; J720 Dental, Stratasys, Israel) to fabricate a simulated reconstructed mandibular truncatable model using medical resin materials (Surgical Guide, Formlabs, America; MED610, Stratasys, Israel). Subsequently, we pre-bent a titanium plate to match the contour of the reconstruction model and secured it with plastic straps (Fig. 2a). The corresponding titanium plate fixation holes were drilled on the reconstructed model to align with the titanium plate holes. Subsequently, we divided the model into two sections: the mandibular portion and the fibular segment portion (Fig. 2b). The template was individually matched to the truncated model. The titanium plate fixation holes were then transferred to the template following the screw trajectory on the model (Fig. 2c). A metal guide ring was fitted to the screw trajectory of the mandibular and fibular osteotomy guide to improve the drilling direction guidance (Fig. 2d). The MOPITS has been finalized at this stage, encompassing both mandible and fibula templates. By utilizing these holes on the fibula template, surgeons can accurately determine the placement of the pre-bent titanium plate, allowing for the shaping of the fibula segment before the pedicle is cut. If the patient's condition allows, simultaneous hydrophilic implant placement can be performed using a customized pioneer drill guide template (Supplemental Digital Content 1).

### Surgical procedure

**Fig. 3:**
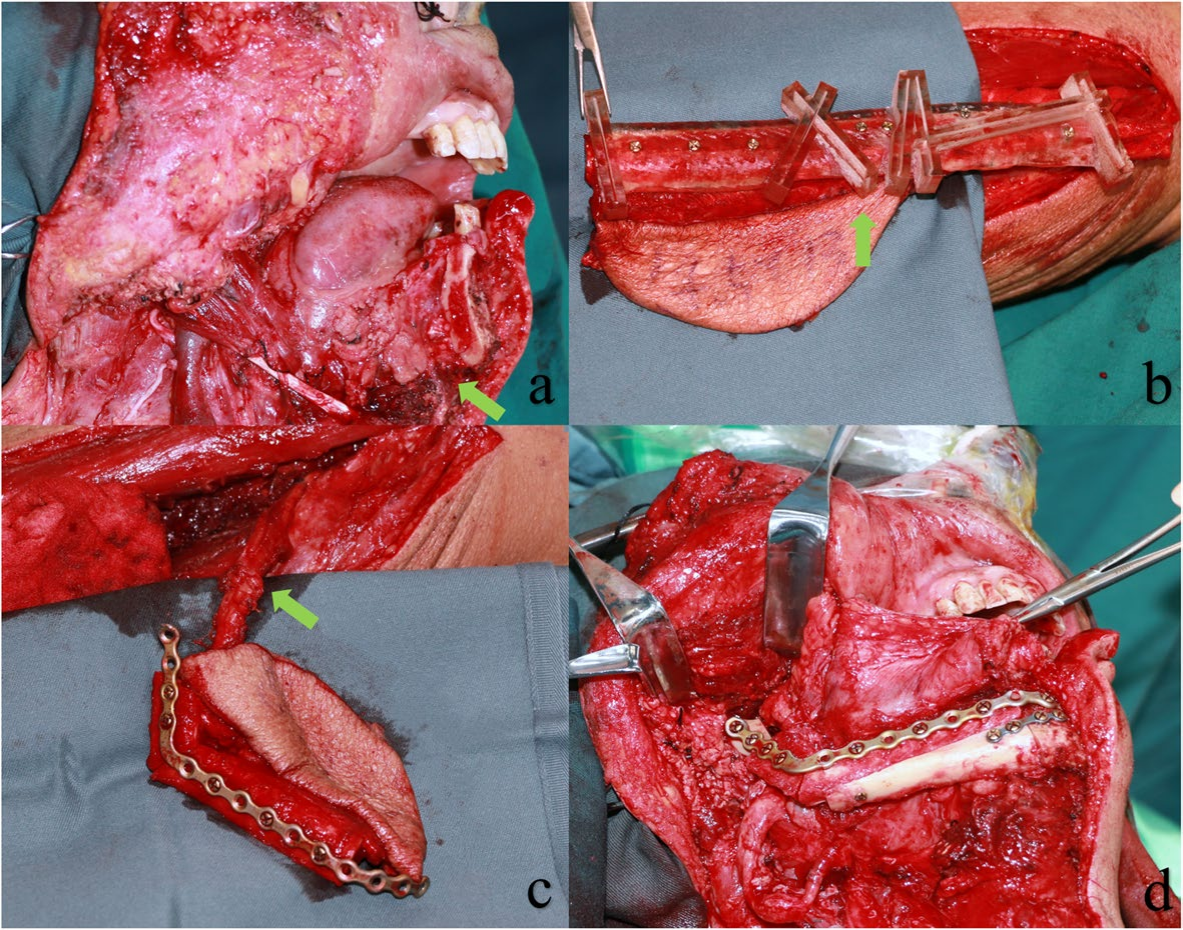
Surgical procedure for reconstruction of mandibular defects with MOPITS guided fibula flap: **a** Mandibular defects; **b** Fabrication of the fibular segment under the guidance of MOPITS. The arrow points to the fibular template. **c** Fixation of the fibular segment with a pre-bent titanium plate. The arrow points to the vascular pedicle. **d** Fixing the fibular flap to the remaining part of the mandible to complete the mandibular reconstruction

A two-team surgical approach was employed for all patients. The cephalic team exposed the mandibular lesion following the preoperative plan and then resected it under the guidance of a resection template (Fig. 3a). Simultaneously, the second surgical team focused on harvesting and shaping the fibular flap. The surgeon exposed the fibula, 6–8 cm from the external ankle joint, as a starting point for osteotomy to maintain ankle stability. A sterilized template was placed on the surface of the fibula, and a titanium screw punch was used to prepare a screw trajectory (Fig. 3b). A titanium screw was then used to secure the plate onto the designated fibular segment. The segment was subsequently cut using a guided cutter slot, and the screw was removed. Then the titanium plate is fixed to the fibular segment in one piece, following the same path as the nail (Fig. 3c). Once the recipient area is prepared, vascular dissection is conducted. The fibula segment, fixed by the titanium plate, is secured to the remaining mandible with titanium screws (Fig. 3d). Finally, the vessels were anastomosed under a microscope, and the skin paddle was trimmed and sutured for stabilization.

### Postoperative evaluation

**Fig. 4:**
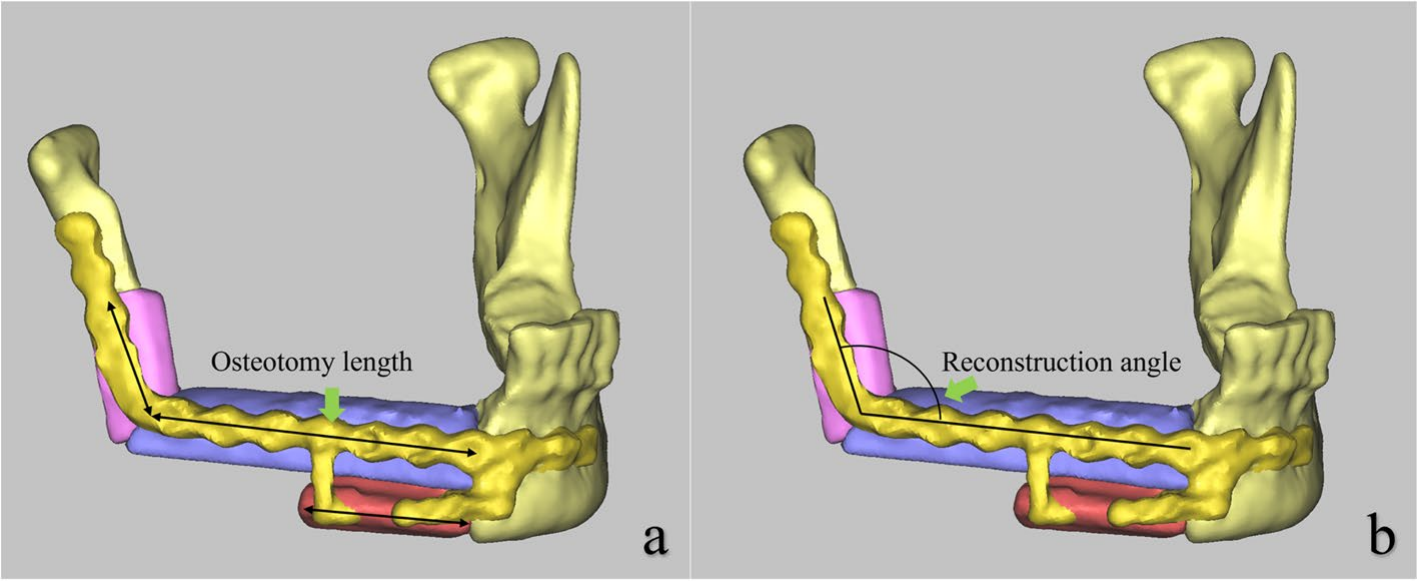
Postoperative accuracy study: **a** Measurement of the length of the osteotomy segment; **b** Measurement of the angle of adjacent segments

All patients underwent CBCT at 2 weeks postoperatively and subsequently every 3 months. The first postoperative CT data of the patients were imported into Proplan to measure the deviation. A line connecting the midpoints of the two ends of the bone segment was selected to measure the length of the osteotomy. These segments' preoperative designed length and the actual osteotomy length were measured, and the absolute difference between the preoperative and postoperative measurements was calculated as the osteotomy error. In cases where multiple fibular segments were included in a single layer, the angles between adjacent segments were measured preoperatively and postoperatively, and the difference between these measurements represented the reconstruction angular error (Fig. 4). To evaluate the symmetry of the patient's mandibular reconstruction, the angle of the mandibular angle was measured bilaterally. A ratio closer to 1 indicated better symmetry in appearance [13]. Additional evaluation outcomes included surgical results based on postoperative follow-up, aesthetic contouring results, and patient satisfaction. Statistical tests were performed using the IBM SPSS Statistics20 package (IBM Corp, Armonk, NY, USA). Continuous values were expressed as mean ± standard deviation (SD). Comparisons were conducted using Student’s t-test for continuous quantitative values. *p* < 0.05 was considered significant.

## Results

**Fig. 5:**
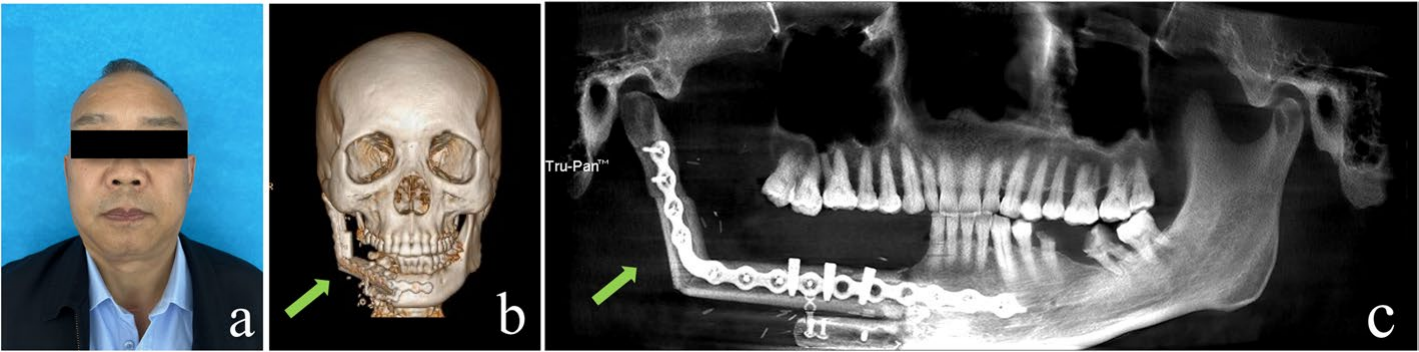
Post-operative follow-up image of a 54-year-old male with SCC of the right mandible gingiva: **a** Post-operative photo; **b** Post-operative CT; **c** post-operative CBCT. The arrow points to the reconstructed mandibular portion of the fibula flap

All procedures were completed with the titanium plates and nails, resulting in mandibular reconstruction following the preoperative design. During the same operation, four patients underwent implant placement, which had an average operative time of 487.25 ± 60.84 min, while the remaining malignant tumor patients had an average operative time of 397.18 ± 73.09 min. All patients received postoperative nasogastric feeding for 3–5 days, and the tube was successfully removed. Two patients exhibited postoperative neck fluid infections, successfully treated with puncture aspiration and antibiotics. The average length of postoperative hospital stay for all patients was 12.67 ± 3.15 days. The titanium plate and a single free bone segment were removed three months after surgery due to a localized infection in one patient. All the fibular segments of the remaining patients survived well. Follow-up evaluations were conducted at intervals ranging from 6 to 35 months post-operation. One patient received postoperative radiotherapy and immunotherapy, 3 patients received postoperative radiotherapy, and the remaining patients did not receive tumor-related adjuvant therapy. One patient with osteosarcoma had lung metastasis during the follow-up period. The remaining patients had no new lymph node or distant metastasis, and all patients survived. Two patients who underwent simultaneous implant placement achieved exposure of implant site and placement of healing abutment six months following the surgical procedure. Finally, the denture was worn successfully. Implant restoration treatment was not provided to the remaining patients due to economic considerations, lack of willingness, and radiotherapy response. All patients exhibited favorable cosmetic and articulation functional outcomes during this period and expressed satisfaction with the surgical results. Postoperative follow-up photograph and CBCT of one patient are shown in Fig. 5.

Fifteen patients underwent procedures involving 2–4 fibular segments, averaging 3 segments per procedure. The average preoperative designed fibular segment length was 44.59 ± 16.20 mm, while the average postoperative actual fibular segment length was 43.91 ± 16.18 mm, resulting in an osteotomy error of 1.01 ± 0.90 mm. The average preoperative designed fibular segment angle was 117.70 ± 10.04°, while the average postoperative actual fibular segment angle was 117.93 ± 10.26°, resulting in a reconstruction angular error of 1.70 ± 1.41°. A comparison was made between pre- and postoperative data, resulting in a *p*-value of > 0.05, which indicates no significant difference. In the symmetry test, the mean preoperative bilateral mandibular angle ratio was 1.01 ± 0.06, and the mean postoperative value was 1.02 ± 0.05.

## Discussion

In 1975, Taylor [14] first introduced the free fibula flap, and Hidalgo [15] reported the first mandibular reconstruction with the fibula in 1989, using multiply defined osteotomies to accurately recreate the shape of the mandible. It has become the first choice for mandibular reconstruction. The utility and techniques of mandible reconstruction using vascularized fibula flaps have significantly advanced over the past two decades [16].

The main challenges associated with the fibula flap procedure are the shaping of the fibula and the positioning of the titanium plate. Prolonged operational time spent on these aspects increases the risk of necrosis, infection, and hematoma of the fibular flap. Therefore, a precise preoperative design of the osteotomy plan, facilitated by digital software, is crucial to minimize potential damage to the fibula flap. Surgical navigation and templates are the two main methods for accurately translating virtual surgical plans into the operating room [17]. Surgical navigation has been proven to be a powerful tool that enables the precise execution of surgical plans. The mandible's mobility poses a challenge in utilizing navigation systems for maxillofacial surgery. In addition, the relatively high cost and technical requirements restrict its application in clinical practice. Surgical templates are based on CAD/CAM and rapid prototyping technology. Tumor resection and fibular osteotomy templates have been extensively utilized in mandibular reconstruction with vascularized fibula flaps [18–21].

To enhance the precision and efficiency of the procedure, certain physicians have utilized pre-bent titanium plate techniques and screw trajectory transfer techniques [7]. Although these approaches have shown promise, they often require additional scanning of reconstructed models, potentially prolonging the design process. Furthermore, Additionally, these techniques often necessitate the use of further shaping guides [8, 9]. Therefore, in order to simplify the surgical procedure and shorten the design period, we propose an improved method for the fabrication of integrative templates. The method features several key improvements. Firstly, precise preoperative pre-bending of the titanium plate is conducted using a truncatable model. This model enables a visual simulation of the intraoperative situation, facilitating flexible adjustments based on the relative position of the plate and the bone segment. In addition, the method enables simulation of the intraoperative screw fixation position and direction and the screw path is transferred in reverse to the template using a truncatable model. The template fabrication process requires only one preoperative scan, design, and 3D printing, leading to a significantly reduced design cycle. Moreover, the fibula segment can be shaped and fixed before cutting the pedicle, eliminating the need for a separate fibula seating guide, allowing quick and precise seating and reducing ischemic time.

In 2019, Dr. Yang [22] proposed a "surgeon-dominated" approach for designing and fabricating surgical guides, in which surgeons are responsible for creating the schematic design, while engineers provide support in designing guide plates and 3D printing of customized titanium plates. This approach offers a significantly shorter turnaround time of around 4 weeks compared to the current commercially available engineer-dominated approach [23]. However, its widespread adoption remains challenging due to high equipment and technical requirement [24]. Our team has developed a modified design method in which a junior physician completes the schematic design based on the lead surgeon's requirements. Subsequent template printing and screw trajectory transfer can be conducted in the laboratory, fulfilling the surgeon's practical needs during clinical work. The entire design and production process can be completed within 2–3 days with the assistance of engineers. Due to the simple design process and short cycle time, CT data can be utilized within a week after the surgery. In our clinical practice, the extent of lesions in patients with malignant tumors tends to remain stable, enabling us to carry out osteotomy as planned without the need for additional modifications. The cost of the guide plate primarily depends on the type and quantity of material utilized for 3D printing [25]. Our current method requires only one preoperative CT scan, combining computer-aided manufacturing and manual handling, thereby reducing production costs. Table 2 presents the characteristics of various guide plate technologies.

**Table 2:** Characteristics of several modified guide plate techniques

Plate Type	Design and Fabrication	Features and Advantages	Shortcomings
MOPITS	Combined with pre-bent titanium plates and Manual mechanical method to simulate intraoperative situation and achieve screw trajectory transfer	Simple design process; Only need two sets of templates; Enables shaping of the fibular flap prior to cutting the pedicle	Need for manual mechanical processing of the guide plate
CORPPP [13, 14]	Scanning with a CBCT of the model with pre-bent titanium plates fixed and designing positioning holes on the mandible guide plates	Easy access to CBCT scan data	Requires a secondary scan and a CORPPP positioning template is needed for fibular segment shaping
Pre-bent plate-positioning surgical guide system [12]	Scanning with a 3D coordinate measuring machine of the pre-bent titanium plate model and designing positioning holes on the guide plate	Enables shaping of the fibular flap prior to cutting the pedicle	Requires a secondary scan and a Shaping Guide is needed for titanium plate positioning
KLB [11]	Commercially available detachable assembly model, combined with small titanium plate fixation	Can be reused on different patients to save costs	Limited applicability; Inadequate contour effects
Three-Dimensionally Printed Patient-Specific Surgical Plates [17]	Manufactured with high precision through selective laser melting (SLA) technology	High degree of personalization; Simple and convenient fixation of double-layer fibular flaps with common titanium plates	High cost and currently difficult to popularize

Our team has utilised MOPITS technology to treat a total of 40 patients, including malignant tumors, benign tumors and maxillary lesions. The outcomes of the surgical interventions were deemed to be satisfactory. To ensure statistical consistency in surgical duration and reconstruction precision, we chose to include 15 patients with mandibular malignancies that had undergone analogous surgical procedures for our study. Virtual planning and guided surgery are generally acknowledged to have the potential to reduce operation time in microvascular free flap reconstruction [26–29]. In our study, the duration of fibula osteotomy ranged from 10 to 48 min, depending on the segment design. Our template enables preoperative shaping of the fibular flap before cutting the pedicle. The transfer of the fibular segment, fixed with titanium plates, to the mandible can be completed in 3–5 min without additional adjustments. We comprehensively reviewed previous medical records of similar procedures performed by the same surgical team. The results revealed that there were 16 cases where surgical guides without holes were utilized, and the average duration of these procedures was 567.81 ± 33.02 min. Additionally, we identified 15 cases where only jaw guides were used to transfer screw trajectory, with an average procedure duration of 471.40 ± 36.06 min. Although the proficiency of the surgical operation and the team cohesion also contributed to reducing the total length of the procedure, it is evident that MOPITS greatly enhances procedure efficiency. Reducing ischemic time and surgery time is beneficial for flap survival, as well as reducing complications and facilitating postoperative recovery in this reconstructive surgery [30]. In the postoperative follow-up, the incidence of infection and flap survival were not significantly different from other methods.

To ensure the accuracy of the reconstruction, we incorporated a guide slot into the plate design. The guiding slots effectively limit the oscillation and displacement of the saw blade, allowing it to follow the direction of the osteotomy design and significantly reducing saw offset compared to linear cutting grooves. In addition, the screw track of the templates is fitted with a metal guide ring, which effectively prevents the drill bit from losing resin material, thereby better guiding the drilling direction. Our study showed no statistically significant differences between the preoperatively designed osteotomy lengths and fibular segment angles and those measured on the postoperative reconstruction model. This indicates that the composite template can accurately perform the reconstructive procedure following the preoperative design. At present, there are different methods for postoperative accuracy verification in the literature [31], and we selected two important indicators for verification. According to reported data, the mean error of the navigation system in mandibular reconstruction using a free fibular flap ranges from 0.043 to 4.67 mm [26, 32–37], and the average error of conventional templates in this surgery ranges from 1.3 to 10.0 mm [38–44]. Therefore, the accuracy of mandibular reconstruction achieved with MOPITS surpasses that of conventional surgery and is comparable to that of intraoperative real-time guided surgery. Despite utilizing our composite template design, there is still an unavoidable error deflection, which may be due to the fact that the anatomical markings on the fibula are not particularly evident, and there may be slight displacement when the guide is placed on the fibula.

For restoring masticatory function, MOPITS can provide simultaneous implant guidance, reducing the recovery period. Patients who opt out of simultaneous implant restoration can have second-stage implant restoration surgery after 6 months from their initial surgery. This group of patients enrolled in the study constitutes a small number of implant restorations, which may be attributed to economic factors, willingness, radiotherapy response, time, and other factors. The follow-up study aims to track the masticatory function of the patients.

As for limitations, due to the retrospective nature of this study, selection bias could only partially be avoided. Therefore, further prospective studies with larger sample sizes are necessary to substantiate our findings in the future.

## Conclusion

In summary, this study presents an approach that utilizes the MOPITS for accurate preoperative planning and intraoperative execution of fibula flap reconstruction. This promising and reliable technique provides high precision and accuracy, serving as an efficient tool for reconstructive surgery. It offers the possibility to standardize complex surgical procedures. Significantly, this streamlined method is particularly beneficial for surgeons striving to master the procedure of mandibular defect reconstruction using a fibular flap.

### Supplementary Information

**Additional file 1: Supplemental Digital Content.** Intergretive template for fibula reconstruction and implant placement.

## Data Availability

The datasets used and analysed during the current study available from the corresponding author on reasonable request.
