# Supplementary figures and images for: Application of a modified osteotomy and positioning integrative template system (MOPITS) based on a truncatable reconstruction model in the precise mandibular reconstruction with fibula free flap: a pilot clinical study
Source: BMC Oral Health. 2023 Nov 8;23:842. doi: 10.1186/s12903-023-03596-6 (PMC10630995; doi:10.1186/s12903-023-03596-6)

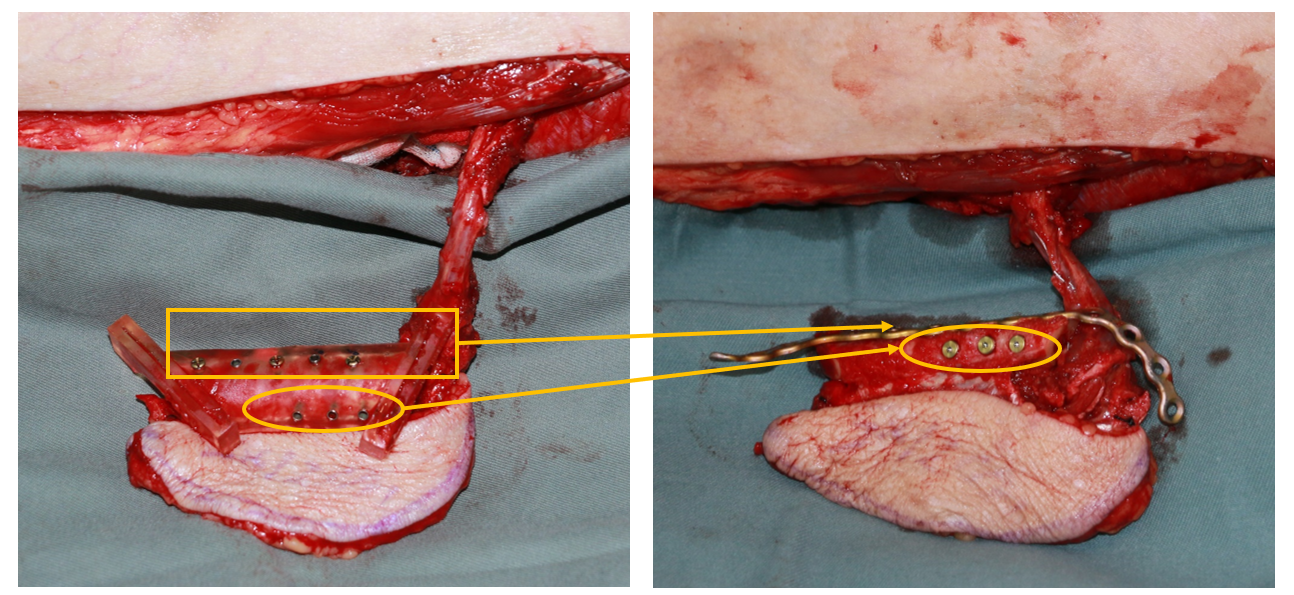

Supplement: Supplementary file 1 — Additional file 1: Supplemental Digital Content. Intergretive template for fibula reconstruction and implant placement. [file 12903_2023_3596_MOESM1_ESM.png]
